# Supplementary material for: Researching the hard-to-reach: a scoping review protocol of digital health research in hidden, marginal and excluded populations
Source: BMJ Open. 2022 Sep 28;12(9):e061361. doi: 10.1136/bmjopen-2022-061361 (PMC9528575; doi:10.1136/bmjopen-2022-061361)
Supplement: Supplementary data [file bmjopen-2022-061361supp001.pdf]

Supplementary Files for "Researching the hard-to-reach: a scoping review protocol of digital health research in hidden, marginal, and excluded populations"

A. Documented search strategy

Contents

I. Summary of search methods and approach
II. Pubmed
III. Cochrane Review
IV. Pysch Info
V. Greenfile
VI. Google Scholar
VII. Grey literature

I. Summary of search methods and approach

- Screening for inclusion of published literature performed by first reviewer from February 1st to March 1st 2022
- Screening for inclusion planned by second reviewer in August 2022
- Data extraction from the first review occurred in parallel following the testing of the data extraction framework by both reviewers.

Summary of search strategy method:

Database/Source	Focus	Article limit per search term combination
Published literature		
PubMed	Health and medical research	None
Cochrane Library	Relevant systematic reviews on digital health	None
PsycINFO	Mental health and social research	None
Google Scholar	All search terms	200
Greenfile	Impact of climate change on health	None
Grey literature		
Websites of key technical agencies (World Health Organization, UNICEF)	Hard-to-reach/marginalization population specific research guidance – digital and non-digital	50
Campbell Collaboration	Social Science	None

II. PubMed [Search performed February 1 2022]

Concept 1: Digital

Keywords: "Digital health research"[TIAB] OR "Digital Survey*"[TIAB] OR "Digital Epidemiology" [TIAB] OR "Online survey*"[TIAB] *OR* "Mobile technology"[TIAB] OR "Health application*"[TIAB] OR "Health app"[TIAB] OR "Interactive Voice Response" [TIAB] OR "SMS" [TIAB] OR "Whatsapp"

[TIAB] OR “Facebook”[TIAB] OR “Twitter”[TIAB] OR “smartphone*”[TIAB] OR “Auto-photography”[TIAB] OR “Video”[TIAB] OR “Photography”[TIAB] OR “Smartwatch*”[TIAB]

MH: “Digital Technology*”[MH] OR “Cell phone”[MH] OR “Mobile Applications”[MH] OR “Social Media”[MH] OR “Digital Technology”[MH] OR “Wearable electronic devices”[MH]

AND

Concept 2: Hard-to-Reach Populations

Keywords: “Hard-to-reach”[TIAB] OR “Marginalized”[TIAB] OR “Refugee*”[TIAB] OR “Migrant*”[TIAB] OR “Vulnerable”[TIAB] OR “Internally Displaced Person*”[TIAB] OR “Slum Resident*”[TIAB] OR “Slumdweller*”[TIAB] OR “Homeless”[TIAB] OR “Housing insecure”[TIAB] OR “Illiterate”[TIAB] OR “Disability”[TIAB] OR “Remote-Rural”[TIAB] OR “Nomadic”[TIAB] OR “Indigenous”[TIAB] OR “Immigrant*”[TIAB] OR “Prisoner*”[TIAB] OR “Low-income”[TIAB] OR “LGTBQ”[TIAB] OR “Injecting drug-users”[TIAB]

MH: “Health Disparity, Minority and Vulnerable Populations”[MH] OR "Homeless Persons"[MH] OR "Opioid-Related Disorders"[MH] OR "Refugees"[MH] OR "Prisoners"[MH] OR "Poverty Areas"[MH] OR "Transients and Migrants"[MH] OR "Health Services for Transgender Persons"[MH] OR "Vulnerable Populations"[MH] OR "Social Vulnerability"[MH] OR "Intellectual Disability"[MH] OR "Disabled Persons"[MH] OR "Social Discrimination"[MH] OR "Persons with Mental Disabilities"[MH]

AND

Concept 3: Health and social-determinants of health

Keywords: “Health”[TIAB] OR “Nutritional Status”[TIAB] OR “Nutrition”[TIAB] OR “Mental Health”[TIAB] OR “Unemployment”[TIAB] OR “Food insecure”[TIAB] OR “Discrimination”[TIAB] OR “Conflict”[TIAB] OR “Natural Disaster”[TIAB] OR “Flooding”[TIAB] OR “Pollution”[TIAB]

MH: "Health"[MH] OR "Social Determinants of Health"[MH] OR "Environmental Pollution"[MH] OR “Natural Disasters”[MH]

Documented Search Strategy

**Final Search –** 4,966, limit to 10 years – 4,499. Search performed Feb 1, 2022	(("Digital health research"[TIAB] OR "Digital Survey*"[TIAB] OR "Digital Epidemiology" [TIAB] OR "Online survey*"[TIAB] OR "Mobile technology"[TIAB] OR "Health application*"[TIAB] OR "Health app"[TIAB] OR "Interactive Voice Response" [TIAB] OR "SMS" [TIAB] OR "Whatsapp" [TIAB] OR "Facebook"[TIAB] OR "Twitter"[TIAB] OR "smartphone*"[TIAB] OR "Auto-photography"[TIAB] OR "Video"[TIAB] OR "Photography"[TIAB] OR "Smartwatch*"[TIAB] OR "Digital Technology*"[MH] OR "Cell phone"[MH] OR "Mobile Applications"[MH] OR "Social Media"[MH] OR "Digital Technology"[MH] OR "Wearable electronic devices"[MH]) AND ("Hard-to-reach"[TIAB] OR "Marginalized"[TIAB] OR "Refugee*"[TIAB] OR "Migrant*"[TIAB] OR "Vulnerable"[TIAB] OR "Internally Displaced Person*"[TIAB] OR "Slum Resident*"[TIAB] OR

	"Slumdweller*"[TIAB] OR "Homeless"[TIAB] OR "Housing insecure"[TIAB] OR "Illiterate"[TIAB] OR "Disability"[TIAB] OR "Remote-Rural"[TIAB] OR "Nomadic"[TIAB] OR "Indigenous"[TIAB] OR "Immigrant*"[TIAB] OR "Prisoner*"[TIAB] OR "Low-income"[TIAB] OR "LGTBQ"[TIAB] OR "Injecting drug-users"[TIAB] OR "Health Disparity, Minority and Vulnerable Populations"[MH] OR "Homeless Persons"[MH] OR "Opioid-Related Disorders"[MH] OR "Refugees"[MH] OR "Prisoners"[MH] OR "Poverty Areas"[MH] OR "Transients and Migrants"[MH] OR "Health Services for Transgender Persons"[MH] OR "Vulnerable Populations"[MH] OR "Social Vulnerability"[MH] OR "Intellectual Disability"[MH] OR "Disabled Persons"[MH] OR "Social Discrimination"[MH] OR "Persons with Mental Disabilities"[MH])) AND ("Health"[TIAB] OR "Nutritional Status"[TIAB] OR "Nutrition"[TIAB] OR "Mental Health"[TIAB] OR "Unemployment"[TIAB] OR "Food insecure"[TIAB] OR "Discrimination"[TIAB] OR "Conflict"[TIAB] OR "Natural Disaster"[TIAB] OR "Flooding"[TIAB] OR "Pollution"[TIAB] OR "Health"[MH] OR "Social Determinants of Health"[MH] OR "Environmental Pollution"[MH] OR "Natural Disasters"[MH])
1 February 2022<br>• Pubmed search exported to endnote<br>• Endnote export to RIS file<br>• 4,499 references uploaded to Rayyan	

III. Cochrane Reviews – Search performed 1 March 2022

Due to the broad reach of the complimentary databases in this review, only Cochrane Reviews were included.

Search Term for Title and Abstract.	Total Cochrane Reviews
"Digital Health"	61
"Digital Technology"	30
"Hard-to-Reach"	3
A total of 91 Cochrane reviews were added to Rayyan.	

IV. PSYCH Info – search performed 20 February 2022

According to the methodology, there was no article limit, although only articles from 2012 were included.

Search Terms combinations	Total articles
(Digital) OR (Mobile technology)	86,159
(Vulnerable) OR (Hard-to-Reach)	89,864

(Health) OR (Climate)	1,963,823
**Final Search Strategy:** “Digital or Mobile technology AND vulnerable or hard-to-reach AND health or climate”	2,307

V. Google Scholar – Search performed on 28 February 2022

According to the methodology, 200 articles were the limit given the potential for duplication with other databases. Only articles from 2012 were included.

Search Terms combinations	Total articles
Vulnerable “digital health”	200

VI. Greenfile – Search performed on 20 February 2022

Search Terms combinations	Total articles
“vulnerable populations or groups or people” AND “digital technology or web technology or software technology or virtual technology”	17

Duplications

Following the compilation of all database results, 401 duplicates were identified by Endnote and Rayyan.

VII. Grey Literature

A grey literature search will focus on guidelines, reviews and commentaries related to the progress of standard setting and best practice for digital research inclusion. The review will include sector Guidance issued from the following institutions: the World Health Organisation, UNICEF and the Campbell Collaboration. References of key studies included in the review will also be considered. The articles identified in the grey literature search will be used to inform the data extraction framework and the analysis. This work is ongoing as of July 2022, informed by the feedback of the reviewers.

Planned search strategy for grey literature:

Search Terms	Database or Website	Document Limit
“Digital Health”, “Digital Technology”, “Hard-to-Reach”	Cochrane Collaboration	50
“Digital Health”, “Digital Technology”, “Hard-to-Reach”	The World Health Organisation, UNICEF	50
“Digital Health”, “Digital Technology”, “Hard-to-Reach” + “Guidance”	Google	50

B. Summary of inclusion and exclusion for published literature

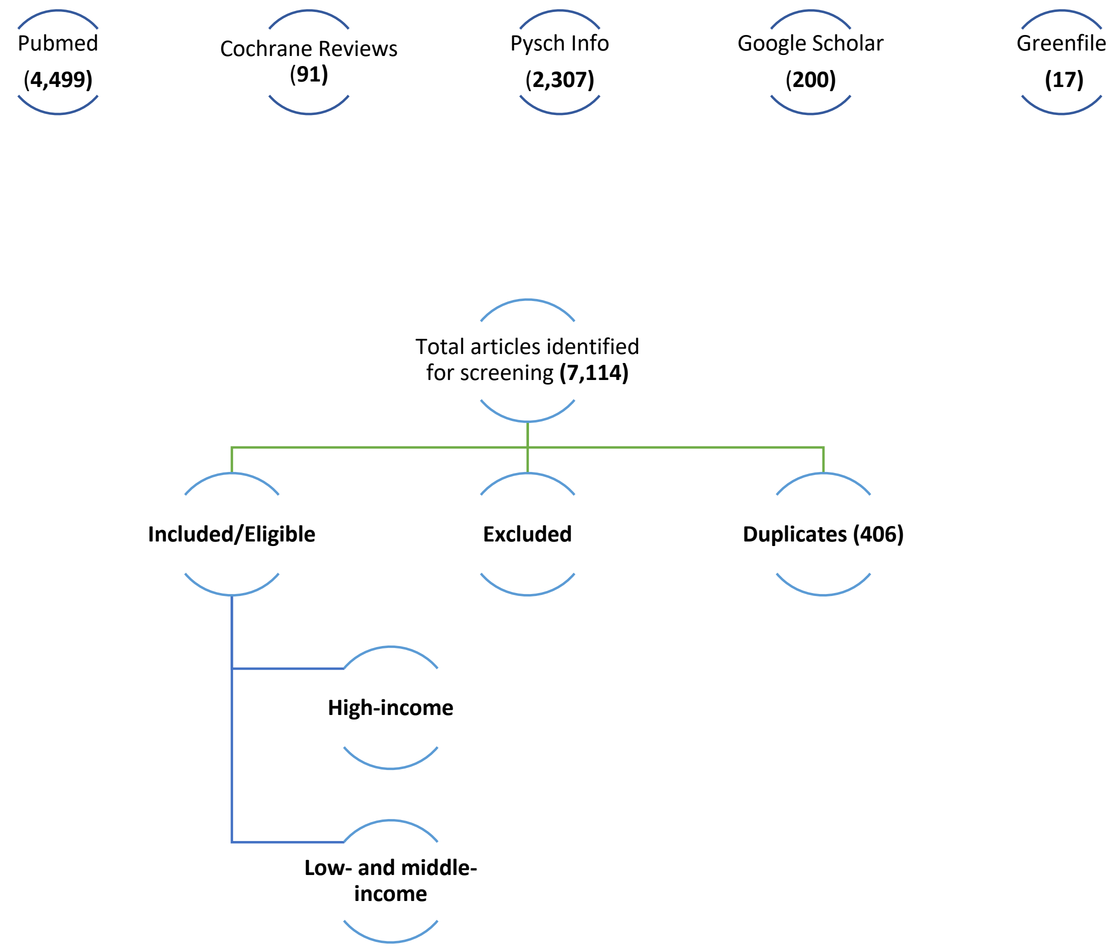

An evidence map was developed to plan the review following guidance of a systematic review by Miake-Lye.(26)

C. Data Extraction Framework

This framework has been adapted from the Chapter 11.3.7.3. of the data extraction guidance from JBI.(25) This is a draft will be adapted and iterated to a final version in line with the iterative process permitted in the Arksey and O' Malley methodological framework and will be documented in the final publication.(27)

Theme	Fields of data collection/categorization
Article source and authorship	Authors<br>Title<br>Journal/Source<br>Year of publication<br>Country<br>High-income country<br>Middle-income country<br>Low-income country
Populations of interest (more than one selection, permitted)	Refugee<br>Migrant<br>Internally Displaced Person<br>Informal Settlement Resident<br>Homeless<br>Low-literacy<br>Disability including physical, developmental, behavioral or emotional and sensory-impaired disorders<br>Remote-rural<br>Nomadic<br>LGBTQ+<br>Low-income<br>Minority<br>Youth *<br>Elderly*<br>Other _____<br>*These two populations are not included alone but with a secondary population. However, due to the presence of a large number of studies related to these populations they have been included for the analysis,
Type of digital research and digital platform (more than one selection, permitted)	Big Data (Machine learning, AI)<br>Digital Survey<br>Digital Epidemiology<br>Online Survey<br>Interactive Voice Response<br>SMS<br>WhatsApp<br>Facebook Survey Twitter<br>App (application, bespoke)<br>Smartphone<br>Auto-photography<br>Video

	Photography<br>Wearable<br>Other ___
Research study focus	Health<br>COVID-19 (y/n)<br>Nutritional status<br>Mental Health<br>Social-determinants of health;(28)<br>• Income and social protection<br>• Education<br>• Unemployment and job insecurity<br>• Working life conditions<br>• Food insecurity<br>• Housing, basic amenities and the environment<br>• Early childhood development<br>• Social inclusion and nondiscrimination<br>• Structural conflict<br>• Access to affordable health services of decent quality<br>Physical environmental determinants of health (air-quality, pollution)<br>Climate-related factors such as flooding and heat
Research category	Qualitative<br>Quantitative
Methods and limitations	Recruitment (multiple selections allowed)<br>• Social Media<br>• In-person<br>• snowball sampling<br>• respondent-driven sampling (RDS)<br>• indigenous field-worker sampling (IFWS)<br>• facility-based sampling (FBS)<br>• targeted sampling (TS)<br>• time-location (space) sampling (TLS)<br>• conventional cluster sampling (CCS)<br>• capture re-capture sampling (CR) (19)<br>• Other<br><br>Response rate<br>Use of incentives<br>Anonymity<br>Ethics<br>Presence of community engagement
Quality	Considerations for a rapid quality review of included studies will be developed based on checklists from Joanna Briggs Institute (JBI) (https://jbi.global/critical-appraisal-tools)
