# Supplementary material for: Researching the hard-to-reach: a scoping review protocol of digital health research in hidden, marginal and excluded populations
Source: BMJ Open. 2022 Sep 28;12(9):e061361. doi: 10.1136/bmjopen-2022-061361 (PMC9528575; doi:10.1136/bmjopen-2022-061361)
Supplement: Author's manuscript [file bmjopen-2022-061361.draft_revisions.pdf]

BMJ Open

BMJ Open is committed to open peer review. As part of this commitment we make the peer review history of every article we publish publicly available.

When an article is published we post the peer reviewers' comments and the authors' responses online. We also post the versions of the paper that were used during peer review. These are the versions that the peer review comments apply to.

The versions of the paper that follow are the versions that were submitted during the peer review process. They are not the versions of record or the final published versions. They should not be cited or distributed as the published version of this manuscript.

BMJ Open is an open access journal and the full, final, typeset and author-corrected version of record of the manuscript is available on our site with no access controls, subscription charges or pay-per-view fees (http://bmjopen.bmj.com).

If you have any questions on BMJ Open's open peer review process please email info.bmjopen@bmj.com

BMJ Open

Researching the hard-to-reach: a scoping review protocol of digital health research in hidden, marginal, and excluded populations

Journal:	*BMJ Open*
Manuscript ID	bmjopen-2022-061361
Article Type:	Protocol
Date Submitted by the Author:	27-Jan-2022

Complete List of Authors:	Belt, Rachel; University of Oxford, Nuffield Department of Women's & Reproductive Health<br>Rahimi, Kazem ; University of Oxford Nuffield Department of Women's & Reproductive Health<br>Cai, Samuel; University of Leicester, Centre for Environmental Health and Sustainability, Department of Health Sciences

Keywords:	PUBLIC HEALTH, Health informatics < BIOTECHNOLOGY & BIOINFORMATICS, Information technology < BIOTECHNOLOGY & BIOINFORMATICS

SCHOLARONE™
Manuscripts

Researching the hard-to-reach: a scoping review protocol of digital health research in hidden, marginal, and excluded populations

Research protocol

Authors:

Corresponding author:
Rachel Belt (0000-0002-0078-1236)
University of Oxford
Nuffield Department of Women's & Reproductive Health
1st Floor, Hayes House, 75 George Street
Oxford, OX1 2BQ, United Kingdom
Rachel.belt@wrh.ox.ac.uk

Co-authors
Kazem Rahimi (0000-0002-4807-4610)
Nuffield Department of Women's & Reproductive Health
1st Floor, Hayes House, 75 George Street
Oxford, OX1 2BQ, United Kingdom
University of Oxford
kazem.rahimi@wrh.ox.ac.uk

Samuel Cai (0000-0003-1601-2199)
Centre for Environmental Health and Sustainability,
Department of Health Sciences,
University Road, LE1 7RH, Leicester, United Kingdom
University of Leicester
yc368@leicester.ac.uk

Word Count 3,761, including supplementary files

For peer review only

ABSTRACT

Introduction

There is significant growth in the use of digital health technology and research methods, further driven by the COVID-19 pandemic. Challenges exist in traditional research methods in reaching hidden, marginalized, and excluded populations. This scoping review aims to map published evidence on and improve understanding of the application of digital research methods to hard-to-reach communities including identification of methods, benefits and potential challenges, and ethical concerns.

Methods and analysis

This review will follow the Arksey and O' Malley methodological framework for scoping reviews. The framework for the review will employ updated methods developed by the Joanna Briggs Institute (JBI) including the PRISMA ScR Checklist. PubMed, the Cochrane Library, PsycINFO, Google Scholar and Greenfile are the identified databases for peer-reviewed quantitative and qualitative studies in-scope of the review. Grey literature focused on guidance and best practice in digital research and hard-to-reach populations will also be searched. Two reviewers will be engaged in the review. After screening the title and abstract to determine eligibility of each article, a thorough full-text review of eligible articles will be conducted using a data extraction framework. Key extracted information will be mapped in tabular and visualized summaries to categorize the breadth of literature and identify key digital methods for use in marginalized populations, their limitations and potential.

Ethics and dissemination

This scoping review does not require ethical approval. The results of the scoping review will consist of peer-reviewed publications, presentations and knowledge mobilization activities including a lay summary posted via social media channels and production of policy brief.

Keywords: Digital health, vulnerable populations, digital research methods

INTRODUCTION

Strengths and limitations of this study

- This review will be the first which considers the intersection of digital health research and marginalized, hidden or excluded populations without a narrow focus on a platform or specific population.
- The rapid expansion in the use of digital health research in the COVID-19 pandemic and the known disparate outcomes of COVID-19 and pandemic-related effects on marginalized populations creates an urgency of understanding if and how digital research includes key populations of interest to ensure data is available to improve health service and inform policy.
- The wide application of health outcomes and the social determinants of health in the inclusion criteria will document existing methods which may have a wide applicability.
- The synthesis of data will be limited to a mapping of methods and categorization and less on the strength of each study due to the variance across the different fields of study and specializations.
- Only peer-reviewed published studies will be included which may omit findings of interest.

For peer review only

Health equity is a common term in policy and research, although significant barriers remain towards achieving health for all. Marginalized, hidden and underserved populations are subject to barriers which affect achievement of equitable health and social welfare outcomes. For this scoping review, hard-to-reach populations are defined as populations who are underserved by health and social services and/or face barriers in achieving equitable health and well-being due to socio-economic determinants of health, discrimination, location, disability or exposure to climate and environmental factors.

Digital research, defined as "the use of online and digital technologies to collect and analyze research data," was increasing pre-pandemic, however, the study of the adaptation of research methods to the context of the pandemic and the quantification of its share in research methods is not widely available or studied.(1) COVID-19 has driven a digital transformation in healthcare, with increases in access to telehealth, the use of Big Data for monitoring the pandemic and the deployment of health communications via social media and other digital platforms.(2–4) The use of online surveys, mobile devices and social media are examples of digital modalities used in digital research and to study the impact and spread of COVID-19. (2,5,6)

Improved understanding of the social determinants of health for hard-to-reach populations is required to achieve the Sustainable Development Goals and its aim to "leave no one behind" and to end the COVID-19 pandemic, which has displayed health and social disparities existing within and between countries.(7–9) Without further review of research methods at the intersection of hard-to-reach populations and digital research, and clarity on barriers to research inclusion, there is a risk that marginalized and vulnerable populations will be further excluded and health disparities, due to the digital divide and other factors, exacerbated.(10,11)

Application of digital research methods represents an opportunity to improve health equity and the inclusion of traditionally hard-to-reach, underserved and marginalized populations in research to inform program design and policy. Previous reviews have documented strategies to improve research with socially disadvantaged groups, reviewed specific digital platforms such as social media and the use of specific health research technologies but none have documented the methods for applying digital research methods to marginalized populations.(2,12–20)

This review will build on the previous reviews including by Bonevski and Shaghaghi, which systematically documented barriers and strategies for research with socially disadvantaged and vulnerable groups. (18,19) This review has considered their findings in the design but will narrow the review to studies where research was conducted, digitally. O'Conner, Helena, Adjekum, Giustini, Whitaker and Edo-Osagie published scoping, literature or systematic reviews of frameworks and analysis of digital health interventions, social media in public health and digital health app development standards. However, these reviews are broader than the methods' application to marginalized or vulnerable populations and the specific barriers or concerns when researching the hard-to-reach. (12–15,17,21) A retrospective analysis of NIH-funded digital health research by Nebeker, confirmed the increase application of social media platforms in the study of hard-to-reach populations. The analysis concluded that guidance to researchers is insufficient in evaluating key concerns around the use of social media in research.(22)

The objective of this scoping review is to identify appropriate digital research methods to improve health research inclusivity, documenting both strategies and limitations of digital research methods with marginalized and hidden populations. This review is important to ensure that the growth in digital methods does not leave behind populations of concern due to design

For peer review only - http://bmjopen.bmj.com/site/about/guidelines.xhtml

limitations. The timing of this review is critical due to the risks of the COVID-19 pandemic on already vulnerable populations and the need to reach and include the most vulnerable and marginalized in research across health and social disciplines to improve health equity.

METHODS AND ANALYSIS

A scoping review was chosen due to the broad nature of the subject. In line with the methods developed by Arksey and O' Malley, this review will follow the framework for conducting a scoping study; Stage 1: identifying the research question, Stage 2: identifying relevant studies, Stage 3: study selection, Stage 4: charting the data and Stage 5: collating, summarizing and reporting the results.(23) Detail on each stage is provided.

Stage 1: Identification the research question

The key research questions outlined in *Table 1* provide focus to the aim of the review to improve understanding of how digital research methods are applied to hard-to-reach populations including identification of methods, potential challenges, and ethical concerns. To inform the search for marginalized, excluded and hidden populations which may not be defined by a term, a list of populations of interest have been included in data extraction framework in *Annex A.*

Table 1: Research questions

**Research Questions**
(1) How are digital health research methods applied to marginalized, excluded and hidden populations?
The secondary research questions focus on the strengths and limitations of the identified methods which include;<br>(1) What are digital research methods commonly applied to include or target hard-to-reach populations?<br>(2) What are challenges in recruitment, retention, and response rate in digitally designed studies for hard-to-reach populations?<br>(3) What were identified areas of ethical, safety and anonymity concerns in using digital research platforms for hard-to-reach populations?

Stage 2: Identifying relevant studies

The search strategy aims to be comprehensive in its inclusion of studies across different socio-economic determinants of health and potential marginalized populations but is limited to peer-reviewed and published literature. The example evidence map is available in *Annex B.* The selection of health, social science, mental health, and climate research databases will be employed in this review.(24) The database, description and article limit can be found in *Table 2.* Grey literature will be reviewed to ensure the inclusion of guidance and sector best practice in the analysis and discussion sections of the scoping review. The example search strategy is available in *Annex C.*

Table 2: Databases and Sources

**Database/Source**	**Focus**	**Article limit per search term combination**
<u>Published literature</u>		
PubMed	Health and medical research	None
Cochrane Library	Relevant systematic reviews on digital health	None
PsycINFO	Mental health and social research	None

For peer review only - http://bmjopen.bmj.com/site/about/guidelines.xhtml

Google Scholar	All search terms	200
Greenfile	Impact of climate change on health	None
Grey literature		
Websites of key technical agencies	Hard-to-reach/marginalization population specific research guidance – digital and non-digital	50

A Population, Concept, Context framework (PCC) for this review is illustrated in *Table 3.*(25) Various search terms will be included, namely common hard-to-reach populations, socio-determinants of health and names of various digital platforms and methods. These are in line with the draft data extraction framework. Completed search terms will be documented and shared in the final publication.

Table 3: Population, Concept, Context framework (PCC)

PCC	Definition	Example
Population	Hard-to-Reach	Homeless
Concept	Digital research methods	Online survey
Context	Health and social research	Mental Health

Stage 3: Study Selection

The review has established a wide inclusion criteria. The geographic scope of this research is global but studies are limited to those published in English. Inclusion and exclusion criteria can be found in *Table 4.* Mendeley will be used to store all search results. The first reviewer will screen all titles and abstracts against the criteria. The second reviewer will screen 10% of these to validate the screening process for inclusion and exclusion criteria. The first reviewer will then import the full text of all included documents for detailed analysis.

Table 4: Inclusion and Exclusion Criteria

Theme	Inclusion Criteria	Exclusion Criteria
Geographic	All	None
Time	Since 2012	Before 2012
Language	English	All other languages
Populations	Keywords listed in *Table A* and search strategy in *Annex C*	Digital health studies which are not focused on hard-to-reach/marginalized populations
Type of literature	Published, peer-reviewed literature<br>Sector guidance published in grey literature	Literature which is not peer-reviewed<br>Unpublished literature which is not considered sector guidance including presentations, webinars, and case studies
Digital Application	Digital research approaches including digital data collection, use of apps to collect data, digital surveys, use of social media and other strategies	Digital interventions such as remote consultations, digital health communications, social media health campaigns.

For peer review only - http://bmjopen.bmj.com/site/about/guidelines.xhtml

		Digital information systems which are not part of a research design method will be excluded.

Stage 4: Charting the data
The data charting will occur using a google form collecting the recommended general information about the study according to the guidance for scoping reviews and expanding data collection through a data extraction framework into key study characteristics, including type of digital study, methodology and limitations. The data extraction framework, available in *Annex A* will be tested by both reviewers for accuracy and applicability and applied consistently across all included studies. The first reviewer will extract all data and the second reviewer will review 10% of the articles to check for alignment. If misalignment is identified, the second reviewer will provide a full review of literature. The grey literature search for global guidance and best practice will be performed by the first reviewer.

Stage 5: Collating, summarizing and reporting the results
The data collected in the charting exercise will be analyzed by both reviewers. The digital methods deployed will be documented with particular focus to how the method enabled inclusivity of the hard-to-reach population and followed sector guidance for ethical research. The limitations of the studies will be analyzed with a focus on research strength, ethics, replicability, and feasibility. Additional analysis will include the identification of key research gaps and future research priorities at the intersection of digital health research and marginalized populations.

PATIENT AND PUBLIC INVOLVEMENT
No patient involved.

ETHICS AND DISSEMINATION
This scoping review does not require ethical approval as it reviews already available publications. The results of the scoping review will consist of peer-reviewed publications, presentations and knowledge mobilization activities including a lay summary posted via social media channels and production of policy brief.

AUTHOR AFFILIATIONS:
University of Oxford
Nuffield Department of Women's & Reproductive Health
1st Floor, Hayes House, 75 George Street
Oxford, OX1 2BQ, United Kingdom

University of Leicester
Centre for Environmental Health and Sustainability,
Department of Health Sciences,
University Road, LE1 7RH, Leicester, United Kingdom
University of Leicester

AUTHORS' CONTRIBUTIONS:
RB: conceived the initial idea, designed the review, drafted the manuscript. KR and SC: provided inputs to the study protocol and revised manuscript. All authors approved the manuscript for submission.

FUNDING STATEMENT:

For peer review only

This research was funded by the PEAK Urban programme, UKRI's Global Challenge Research Fund, Grant Ref: ES/P011055/1; supported by the National Institute for Health Research (NIHR) Oxford Biomedical Research Centre (BRC), the Oxford Martin school, University of Oxford and the British Heart Foundation grant ref: PG/18/65/33872. The views expressed are those of the authors and not necessarily those of the OMS, the UK National Health Service (NHS), the NIHR or the Department of Health and Social Care.

The funding organizations had no role in design or conduct of the study; collection, management, analysis, and interpretation of the data; preparation, review, or approval of the manuscript; or decision to submit the manuscript for publication.

SC acknowledges support from National Institute for Health Research (NIHR) Health Protection Research Unit in Environmental Exposures and Health, a partnership between the UK Health Security Agency, the Health and Safety Executive and the University of Leicester. The views expressed are those of the author(s) and not necessarily those of the NHS, the NIHR, the Department of Health and Social Care or the UK Health Security Agency.

COMPETING INTERESTS STATEMENT

The authors note there are no competing interests, financial or personal which have influenced this research.

REFERENCES

1. Snee H., Hine C., Morey Y. et al. Digital Methods as Mainstream Methodology: An Introduction. In: Snee H., Hine C., Morey Y. et al. *Digital Methods for Social Science.* London: Palgrave Macmillan 2016: 1-11
2. Gunasekeran DV, Tseng RMWW, Tham YC, et al. Applications of digital health for public health responses to COVID-19: a systematic scoping review of artificial intelligence, telehealth and related technologies. *Digital Medicine* 2021. https://www.nature.com/articles/s41746-021-00412-9 (accessed 12 Dec 2021)
3. Rodriguez JA, Clark CR, Bates DW. Digital Health Equity as a Necessity in the 21st Century Cures Act Era. *JAMA : the journal of the American Medical Association*. 323(23):2381–2. doi:10.1001/jama.2020.7858 (accessed 12 Dec 2021)
4. Keesara S, Jonas A, Schulman K. Covid-19 and Health Care's Digital Revolution. *New England Journal of Medicine.* 2020 Jun 4 382(23):e82. https://www.nejm.org/doi/full/10.1056/NEJMp2005835 (accessed 7 Jan 2022)
5. Whitelaw S, Mamas MA, Topol E, C Van Spall HG. Applications of digital technology in COVID-19 pandemic planning and response. *The Lancet Digital Health* August 01 2020; Volume 2 Issue 8, E4345-E440. https://www.thelancet.com/journals/landig/article/PIIS2589-7500(20)30142-4/fulltext (accessed 12 Dec 2021)
6. Hlatshwako TG, Shah SJ, Kosana P, Adebayo E et al. Online health survey research during COVID-19. *The Lancet Digital health* 2021 Feb 3(2):e76–7. http://www.ncbi.nlm.nih.gov/pubmed/33509387 (accessed 8 Jan 2022)
7. "The 17 Goals: Sustainable Development" https://sdgs.un.org/goals (accessed 7 Jan 2022)

For peer review only - http://bmjopen.bmj.com/site/about/guidelines.xhtml

8. Mude W, Oguoma VM, Nyanhanda T et al. Racial disparities in COVID-19 pandemic cases, hospitalisations, and deaths: A systematic review and meta-analysis. *Journal of Global Health*. 2021 11: 05015. https://www.ncbi.nlm.nih.gov/pmc/articles/PMC8248751/ (accessed 7 Jan 2022)
9. Jacques A, Ambrose H. Inequities During COVID-19. *Pediatrics* 2020 Aug;146(2):e202015012020 https://doi.org/10.1542/peds.2020-1501 (accessed 7 Jan 2022)
10. Nebeker C, Torous J, Bartlett Ellis RJ. Building the case for actionable ethics in digital health research supported by artificial intelligence. *BMC Medicine* 2019 Jul 17:1–7. https://bmcmedicine.biomedcentral.com/articles/10.1186/s12916-019-1377-7 (accessed 11 Dec 2022)
11. Whitelaw S, Mamas MA, Topol E, et al. Applications of digital technology in COVID-19 pandemic planning and response. *The Lancet* Volume 2, Issue 8 2020, E435-E440, August 2 2020 https://www.thelancet.com/journals/landig/article/PIIS2589-7500(20)30142-4/fulltext (accessed 7 Jan 2022)
12. O'Connor, S., Hanlon, P., O'Donnell, C.A. et al. Understanding factors affecting patient and public engagement and recruitment to digital health interventions: a systematic review of qualitative studies. *BMC Medical Informatics and Decision Making* 16, 120 (2016). https://doi.org/10.1186/s12911-016-0359-3 (accessed 12 Dec 2022)
13. Helena M, Velthoven V, Smith J, Wells G, Brindley D. Digital health app development standards: a systematic review protocol. *BMJ Open* 2018 8:22969 https://bmjopen.bmj.com/content/8/8/e022969 (accessed 12 Dec 2021)
14. Giustini D, Ali SM, Fraser M, Kamel Boulos MN. Effective uses of social media in public health and medicine: a systematic review of systematic reviews. *Online J Public Health Informatics.* 2018 Sep 21;10(2):e215 https://pubmed.ncbi.nlm.nih.gov/30349633/ (accessed 12 Dec 2021)
15. Adjekum A, Blasimme A, Vayena E. Elements of Trust in Digital Health Systems: Scoping Review. *Journal Medical Internet Research* 2018;20(12):e11254 https://www.jmir.org/2018/12/e11254 (accessed 12 Dec 2021)
16. Soobiah C, Cooper M, Kishimoto V, et al. Identifying optimal frameworks to implement or evaluate digital health interventions: a scoping review protocol. *BMJ Open* 2020 10:37643. https://bmjopen.bmj.com/content/10/8/e037643 (accessed 12 Dec 2021)
17. Edo-Osagie O, de La Iglesia B, Lake I, Edeghere O. A scoping review of the use of Twitter for public health research. *Computers in Biology and Medicine.* 2020 Jul 1;122:103770. https://www.sciencedirect.com/science/article/pii/S0010482520301426 (accessed 12 Dec 2021)
18. Bonevski B, Randell M, Paul C, Chapman K, Twyman L, Bryant J, et al. Reaching the hard-to-reach: a systematic review of strategies for improving health and medical research with socially disadvantaged groups *BMC Medical Research Methodology* 25 March 2014. Available from: http://www.biomedcentral.com/1471-2288/14/42 (accessed 12 Dec 2021)
19. Shaghaghi A, Bhopal RS, Sheikh A. Approaches to Recruiting "Hard-To-Reach" Populations into Re-search: A Review of the Literature. *Health Promotion Perspectives.*

For peer review only

For peer review only - http://bmjopen.bmj.com/site/about/guidelines.xhtml

2011;1(2):86–94. https://www.ncbi.nlm.nih.gov/pmc/articles/PMC3963617/ (accessed 12 Dec 2021)

20. Thunberg S, Arnell L. Pioneering the use of technologies in qualitative research – A research review of the use of digital interviews. *International journal of social research* methodology. June 2021 :1–12. https://www.researchgate.net/publication/352055837_Pioneering_the_use_of_technologies_in_qualitative_research_-_A_research_review_of_the_use_of_digital_interviews (accessed 12 Dec 2021)
21. Whitaker C, Stevelink S, Fear N. The use of Facebook in recruiting participants for health research purposes: A systematic review. *Journal of medical Internet research.* 2017 August 28:19(8):e290–e290. https://pubmed.ncbi.nlm.nih.gov/28851679/ (accessed 17 Dec 2021)
22. Nebeker C, Dunseath SE, Linares-Orozco R. A retrospective analysis of NIH-funded digital health research using social media platforms. *Digital Health.* 2020 January 21;6:2055207619901085 https://pubmed.ncbi.nlm.nih.gov/32030195/ (accessed 12 Dec 2021)
23. Arksey H, O'Malley L. Scoping studies: towards a methodological framework. *International journal of social research methodology*. 23 Feb 20078(1):19–32. https://www.tandfonline.com/doi/abs/10.1080/1364557032000119616 (accessed 12 Dec 2021)
24. Bramer, W.M., Rethlefsen, M.L., Kleijnen, J. et al. Optimal database combinations for literature searches in systematic reviews: a prospective exploratory study. *Syst Rev* 6, 245 (2017). https://doi.org/10.1186/s13643-017-0644-y (accessed 11 Jan 2022)
25. Peters MDJ, Godfrey C, McInerney P, Munn Z, Tricco AC, Khalil, H. Chapter 11: Scoping Reviews (2020 version). In: Aromataris E, Munn Z (Editors). *JBI Manual for Evidence Synthesis,* JBI, 2020. https://doi.org/10.46658/JBIMES-20-12 (accessed 11 Jan 2022)
26. Miake-Lye, I.M., Hempel, S., Shanman, R. et al. What is an evidence map? A systematic review of published evidence maps and their definitions, methods, and products. *Syst Rev* 5, 28 (2016). https://doi.org/10.1186/s13643-016-0204-x (accessed 11 Jan 2022)
27. Arksey H, O'Malley L. Scoping studies: Towards a methodological framework. *International Journal of Social Research Methodology: Theory and Practice*. 2005 Feb;8(1):19–32. DOI: 10.1080/1364557032000119616 (accessed 12 Dec 2021)
28. "Social determinants of health" The World Health Organization. Available from: https://www.who.int/health-topics/social-determinants-of-health#tab=tab_1 (accessed 12 Dec 2021)

For peer review only - http://bmjopen.bmj.com/site/about/guidelines.xhtml

SUPPLEMENTARY MATERIAL

Annex A: Draft Data Extraction Framework

This framework has been adapted from the Chapter 11.3.7.3. of the data extraction guidance from JBI.(25) This is a draft will be adapted and iterated to a final version in line with the iterative process permitted in the Arksey and O' Malley methodological framework and will be documented in the final publication.(27)

Theme	Fields of data collection/categorization
Article source and authorship	Objectives<br>Authors<br>Title<br>Source<br>Year of publication<br>Country
Populations of interest	Hard-to-reach<br>Marginalized<br>Refugee<br>Migrant<br>Internally Displaced Person<br>Slum resident<br>Informal Settlement Resident<br>Homeless<br>Low-literacy
	Disability including physical, developmental, behavioral or emotional and sensory-impaired disorders<br>Hidden population<br>Remote-rural<br>Nomadic
Type of digital research and digital platform	Digital Research<br>Digital Survey<br>Digital Epidemiology<br>Online Survey<br>Interactive Voice Response<br>SMS<br>WhatsApp<br>Facebook Survey Twitter<br>App (application)<br>Smartphone<br>Auto-photography<br>Video<br>Photography<br>Wearables

For peer review only

Research study focus	Health<br>Nutritional status<br>Mental Health<br>Social-determinants of health;(28)<br>- Income and social protection<br>- Education<br>- Unemployment and job insecurity<br>- Working life conditions<br>- Food insecurity<br>- Housing, basic amenities and the environment<br>- Early childhood development<br>- Social inclusion and nondiscrimination<br>- Structural conflict<br>- Access to affordable health services of decent quality<br>Physical environmental determinants of health (air-quality, pollution)<br>Climate-related factors such as flooding and heat
Research category	Qualitative Quantitative
Methods and limitations	Retention<br>Recruitment<br>- snowball sampling<br>- respondent-driven sampling (RDS)<br>- indigenous field-worker sampling (IFWS)
	- facility-based sampling (FBS)<br>- targeted sampling (TS)<br>- time-location (space) sampling (TLS)<br>- conventional cluster sampling (CCS)<br>- capture re-capture sampling (CR) (19)<br>Response rate<br>Use of incentives<br>Anonymity<br>Ethics<br>Cost<br>Replicability<br>Sample size<br>Discussion Section: study limitations

Annex B: Evidence Map Template

An evidence map was developed to plan the review following guidance of a systematic review by Miake-Lye.(26)

For peer review only

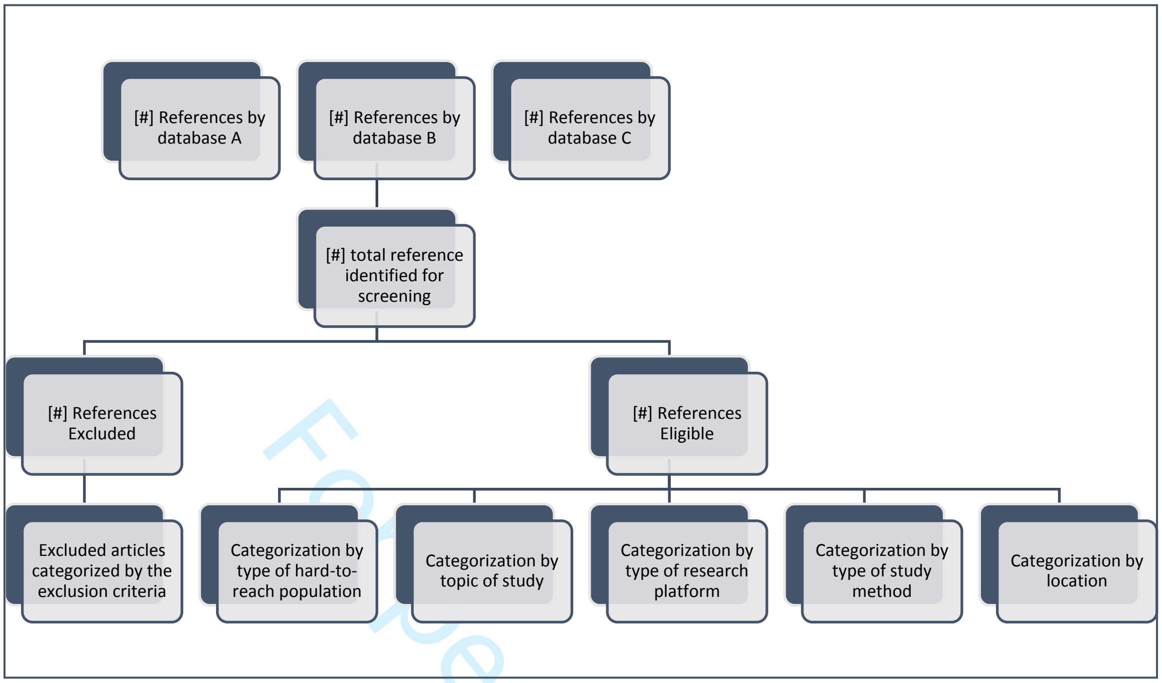

Annex C: Example Search Strategy

I. Pubmed

<u>Concept 1: Digital</u>

Keyword: "Digital health research"[tiab] OR "Digital Survey*"[tiab] OR "Digital Epidemiology" [tiab] OR "Online survey*"[tiab] *OR* "Mobile technology"[tiab] OR "Health application*"[tiab] OR "Health app"[tiab] OR "Interactive Voice Response" [tiab] OR "SMS" [tiab] OR "Whatsapp" [tiab] OR "Facebook"[tiab] OR "Twitter"[tiab] OR "Smartphone*"[tiab] OR "Auto-photography"[tiab] OR "Video"[tiab] OR "Photography"[tiab] OR "Smartwatch*"[tiab]

MeSH: "Digital Technology*"[MH] OR "Cell phone"[MH] OR "Mobile Applications"[MH] OR "Social Media"[MH] OR "Digital Technology"[MH] OR "Wearable electronic devices"[MH]

And,

<u>Concept 2: Hard-to-Reach Populations</u>

Keywords: "Hard-to-reach"[TIAB] OR "Marginalized"[TIAB] OR "Refugee*"[TIAB] OR "Migrant*"[TIAB] OR "Vulnerable"[TIAB] OR "Internally Displaced Person*"[TIAB] OR "Slum Resident*"[TIAB] OR "Slumdweller*"[TIAB] OR "Homeless"[TIAB] OR "Housing insecure"[TIAB] OR "Illiterate"[TIAB] OR "Disability"[TIAB] OR "Remote-Rural"[TIAB] OR "Nomadic"[TIAB] OR "Indigenous"[TIAB] OR "Immigrant*"[TIAB] OR "Prisoner*"[TIAB]

MeSH: "Health Disparity, Minority and Vulnerable Populations"[MH] OR "Homeless Persons"[MeSH] OR "Opioid-Related Disorders"[MeSH] OR "Refugees"[MeSH] OR

For peer review only

"Prisoners"[MeSH] OR "Poverty Areas"[MeSH] OR "Transients and Migrants"[MeSH] OR "Health Services for Transgender Persons"[MeSH] OR "Vulnerable Populations"[MeSH] OR "Social Vulnerability"[MeSH] OR "Intellectual Disability"[MeSH] OR "Disabled Persons"[MeSH] OR "Social Discrimination"[MeSH] OR "Persons with Mental Disabilities"[MeSH]

And,

Concept 3: Health and social-determinants of health

MeSH: "Health"[MH] OR "Social Determinants of Health"[MH] OR "Environmental Pollution"[MH] OR "Natural Disaster"[MH]

For peer review only

Preferred Reporting Items for Systematic reviews and Meta-Analyses extension for Scoping Reviews (PRISMA-ScR) Checklist

SECTION	ITEM	PRISMA-ScR CHECKLIST ITEM	REPORTED ON PAGE #
**TITLE**			
Title	1	Identify the report as a scoping review.	1
**ABSTRACT**			
Structured summary	2	Provide a structured summary that includes (as applicable): background, objectives, eligibility criteria, sources of evidence, charting methods, results, and conclusions that relate to the review questions and objectives.	1
**INTRODUCTION**			
Rationale	3	Describe the rationale for the review in the context of what is already known. Explain why the review questions/objectives lend themselves to a scoping review approach.	2, 3
Objectives	4	Provide an explicit statement of the questions and objectives being addressed with reference to their key elements (e.g., population or participants, concepts, and context) or other relevant key elements used to conceptualize the review questions and/or objectives.	3
**METHODS**			
Protocol and registration	5	Indicate whether a review protocol exists; state if and where it can be accessed (e.g., a Web address); and if available, provide registration information, including the registration number.	3
Eligibility criteria	6	Specify characteristics of the sources of evidence used as eligibility criteria (e.g., years considered, language, and publication status), and provide a rationale.	5
Information sources*	7	Describe all information sources in the search (e.g., databases with dates of coverage and contact with authors to identify additional sources), as well as the date the most recent search was executed.	5
Search	8	Present the full electronic search strategy for at least 1 database, including any limits used, such that it could be repeated.	Annex C
Selection of sources of evidence†	9	State the process for selecting sources of evidence (i.e., screening and eligibility) included in the scoping review.	5
Data charting process‡	10	Describe the methods of charting data from the included sources of evidence (e.g., calibrated forms or forms that have been tested by the team before their use, and whether data charting was done independently or in duplicate) and any processes for obtaining and confirming data from investigators.	6
Data items	11	List and define all variables for which data were sought and any assumptions and simplifications made.	NA at this stage
Critical appraisal of individual sources of evidence§	12	If done, provide a rationale for conducting a critical appraisal of included sources of evidence; describe the methods used and how this information was used in any data synthesis (if appropriate).	NA at this stage
Synthesis of results	13	Describe the methods of handling and summarizing the data that were charted.	6

For peer review only

SECTION	ITEM	PRISMA-ScR CHECKLIST ITEM	REPORTED ON PAGE #
**RESULTS**			
Selection of sources of evidence	14	Give numbers of sources of evidence screened, assessed for eligibility, and included in the review, with reasons for exclusions at each stage, ideally using a flow diagram.	NA at this stage, Draft in Annex B.
Characteristics of sources of evidence	15	For each source of evidence, present characteristics for which data were charted and provide the citations.	NA at this stage
Critical appraisal within sources of evidence	16	If done, present data on critical appraisal of included sources of evidence (see item 12).	"
Results of individual sources of evidence	17	For each included source of evidence, present the relevant data that were charted that relate to the review questions and objectives.	"
Synthesis of results	18	Summarize and/or present the charting results as they relate to the review questions and objectives.	"
**DISCUSSION**			
Summary of evidence	19	Summarize the main results (including an overview of concepts, themes, and types of evidence available), link to the review questions and objectives, and consider the relevance to key groups.	NA at this stage
Limitations	20	Discuss the limitations of the scoping review process.	"
Conclusions	21	Provide a general interpretation of the results with respect to the review questions and objectives, as well as potential implications and/or next steps.	"
**FUNDING**			
Funding	22	Describe sources of funding for the included sources of evidence, as well as sources of funding for the scoping review. Describe the role of the funders of the scoping review.	6

JBI = Joanna Briggs Institute; PRISMA-ScR = Preferred Reporting Items for Systematic reviews and Meta-Analyses extension for Scoping Reviews.

* Where *sources of evidence* (see second footnote) are compiled from, such as bibliographic databases, social media platforms, and Web sites.

† A more inclusive/heterogeneous term used to account for the different types of evidence or data sources (e.g., quantitative and/or qualitative research, expert opinion, and policy documents) that may be eligible in a scoping review as opposed to only studies. This is not to be confused with *information sources* (see first footnote).

‡ The frameworks by Arksey and O'Malley (6) and Levac and colleagues (7) and the JBI guidance (4, 5) refer to the process of data extraction in a scoping review as data charting.

§ The process of systematically examining research evidence to assess its validity, results, and relevance before using it to inform a decision. This term is used for items 12 and 19 instead of "risk of bias" (which is more applicable to systematic reviews of interventions) to include and acknowledge the various sources of evidence that may be used in a scoping review (e.g., quantitative and/or qualitative research, expert opinion, and policy document).

From: Tricco AC, Lillie E, Zarin W, O'Brien KK, Colquhoun H, Levac D, et al. PRISMA Extension for Scoping Reviews (PRISMAScR): Checklist and Explanation. Ann Intern Med. 2018;169:467–473. doi: 10.7326/M18-0850.

For peer review only - http://bmjopen.bmj.com/site/about/guidelines.xhtml

BMJ Open

Researching the hard-to-reach: a scoping review protocol of digital health research in hidden, marginal, and excluded populations

Journal:	*BMJ Open*
Manuscript ID	bmjopen-2022-061361.R1
Article Type:	Protocol
Date Submitted by the Author:	19-Jul-2022
Complete List of Authors:	Belt, Rachel; University of Oxford, Nuffield Department of Women's & Reproductive Health<br>Rahimi, Kazem ; University of Oxford Nuffield Department of Women's & Reproductive Health<br>Cai, Samuel; University of Leicester, Centre for Environmental Health and Sustainability, Department of Health Sciences
**Primary Subject Heading**:	Global health
Secondary Subject Heading:	Health informatics, Health policy
Keywords:	PUBLIC HEALTH, Health informatics < BIOTECHNOLOGY & BIOINFORMATICS, Information technology < BIOTECHNOLOGY & BIOINFORMATICS

SCHOLARONE™
Manuscripts

Researching the hard-to-reach: a scoping review protocol of digital health research in hidden, marginal, and excluded populations

Research protocol

Authors:

Corresponding author:
Rachel Belt (0000-0002-0078-1236)
University of Oxford
Nuffield Department of Women's & Reproductive Health
1st Floor, Hayes House, 75 George Street
Oxford, OX1 2BQ, United Kingdom
Rachel.belt@wrh.ox.ac.uk

Co-authors
Kazem Rahimi (0000-0002-4807-4610)
Nuffield Department of Women's & Reproductive Health
1st Floor, Hayes House, 75 George Street
Oxford, OX1 2BQ, United Kingdom
University of Oxford
kazem.rahimi@wrh.ox.ac.uk

Yutong Samuel Cai (0000-0003-1601-2199)
Centre for Environmental Health and Sustainability,
Department of Health Sciences,
University Road, LE1 7RH, Leicester, United Kingdom
University of Leicester
yc368@leicester.ac.uk

Word Count 3,535, excluding supplementary files

For peer review only

ABSTRACT

Introduction

There is significant growth in the use of digital technology and methods in health-related research, further driven by the COVID-19 pandemic. This has offered a potential to apply digital health research in hidden, marginalized, and excluded populations who are traditionally not easily reached due to economic, societal, and legal barriers. To better inform future digital health studies of these vulnerable populations, we proposed a scoping review to comprehensively map published evidence and guidelines on the applications and challenges of digital health research methods to hard-to-reach communities.

Methods and analysis

This review will follow the Arksey and O' Malley methodological framework for scoping reviews. The framework for the review will employ updated methods developed by the Joanna Briggs Institute (JBI) including the PRISMA ScR Checklist. PubMed, the Cochrane Library, PsycINFO, Google Scholar and Greenfile are the identified databases for peer-reviewed quantitative and qualitative studies in-scope of the review. Grey literature focused on guidance and best practice in digital health research and hard-to-reach populations will also be searched, following published protocols. The review will focus on literature published between 01/02/2012 and 01/02/2022. Two reviewers are engaged in the review. After screening the title and abstract to determine eligibility of each article, a thorough full-text review of eligible articles will be conducted using a data extraction framework. Key extracted information will be mapped in tabular and visualized summaries to categorize the breadth of literature and identify key digital methods, including their limitations and potential, for use in hard-to-reach populations.

Ethics and dissemination

This scoping review does not require ethical approval. The results of the scoping review will consist of peer-reviewed publications, presentations and knowledge mobilization activities including a lay summary posted via social media channels and production of policy brief.

Keywords: Digital health, vulnerable populations, digital research methods

For peer review only

For peer review only - http://bmjopen.bmj.com/site/about/guidelines.xhtml

Strengths and limitations of this study

- This review will be the first which considers the intersection of digital health research and marginalized, hidden or excluded populations without a narrow focus on a platform or specific population.
- The rapid expansion in the use of digital health research risks further exacerbation of the digital divide among populations. This review will provide timely information on targeted strategies to promote and improve inclusivity of hard-to-reach populations in digital health research.
- The inclusion criteria for this scoping review is set to be broad, including any digital research on both the environmental and social determinants of health in hard-to-reach populations. This allows a thorough review of existing digital methods used across different sectors and disciplines that may have a wide applicability to hard-to-reach population research.
- The synthesis of data will be limited to a mapping of methods and categorization and less on the strength of each study due to the variance across the different fields of study and specializations.
- The search is limited in publications in English language only.

INTRODUCTION

Globally, health equity is not consistently or systematically applied within or across populations and sub-populations. Marginalized, hidden and underserved populations are subject to barriers which affect achievement of equitable health and social welfare outcomes. Populations residing in informal settlements, for example, face many socioeconomic, environmental, and legal barriers that prohibit their access to health service. For example, they may lack formal government recognition, therefore their health status and needs are not tracked sufficiently by official systems. This poor data presents a formidable challenge for both researchers and policymakers to redress health inequities among these populations.(1)

Digital research, defined as "the use of online and digital technologies to collect and analyze research data," was increasing pre-pandemic.(2) COVID-19 has driven a digital transformation in healthcare, with increases in access to telehealth, the use of Big Data for monitoring the pandemic and the deployment of health communications via social media and other digital platforms.(3–5) The use of online surveys, mobile devices and social media are examples of digital modalities used in digital research and to study the impact and spread of COVID-19.(3,6,7) The speed and breadth of COVID-19 research was impressive, yet commentaries like those of Jung et al. raise concerns regarding the adherence to research quality and standards in terms of study design, data quality and analytical methods.(8) The latest guidance in the area of digital health such as the WHO Digital Health Guidelines and the Digital Health Equity Framework are opportunities to assess the quality of digital research.(9,10)

Improved understanding of the health for hard-to-reach populations is required to achieve the Sustainable Development Goals and to end the COVID-19 pandemic, which has displayed health and social disparities existing within and between countries.(11–13) For this scoping review, hard-to-reach populations are defined as populations who are underserved by health and social services and/or face barriers in achieving equitable health and well-being due to socio-economic determinants of health, discrimination, location, disability or exposure to climate

For peer review only - http://bmjopen.bmj.com/site/about/guidelines.xhtml

and environmental factors. Without further review of research methods at the intersection of hard-to-reach populations and digital research, and clarity on barriers to research inclusion, there is a risk that these populations will be further excluded and health disparities, partly due to the increasing digital divide, will be exacerbated.(14,15)

Application of digital research methods represents an opportunity to improve health equity and the inclusion of traditionally hard-to-reach, underserved and marginalized populations in research to inform program design and policy. Previous reviews have documented strategies to improve research with socially disadvantaged groups and reviewed specific digital platforms such as social media and the use of specific health research technologies. However, none have systematically documented the methods for applying digital research methods to a hard-to-reach population.(3,16–24)

This review will build on the previous reviews including by Bonevski and Shaghaghi, which systematically documented barriers and strategies for research with socially disadvantaged and vulnerable groups. (22,23) Our scoping review has considered their findings in the design but we will narrow the review to studies in which research was conducted, digitally. O'Conner (16), Helena (17), Adjekum (19), Giustini (18), Whitaker (21,25) and Edo-Osagie(21) published scoping, literature or systematic reviews, or frameworks and analysis of digital health interventions, social media in public health and digital health app development standards. However, these reviews are broader than the scopes in our proposed review, in which we will focus on the methods' application to hard-to-reach populations and the specific barriers or concerns when researching the hard-to-reach. (16–19,21,25) A retrospective analysis of National Institute of Health (NIH)-funded digital health research by Nebeker documented the increased application of social media platforms in the study of hard-to-reach populations. The analysis concluded that guidance to researchers is insufficient in evaluating key concerns around the use of social media in research.(26)

The objective of this scoping review is to identify appropriate digital research methods to improve health research inclusivity, documenting both strategies and limitations of digital research methods with hard-to-reach populations. This review is important to ensure that the growth in digital methods does not leave behind populations of concern due to design limitations. The timing of this review is critical due to the risks of the COVID-19 pandemic on already vulnerable populations and the need to reach and include the most vulnerable and marginalized in research across health and social disciplines to improve health equity.

METHODS AND ANALYSIS

A scoping review was chosen due to the broad nature of the subject. In line with the methods developed by Arksey and O' Malley, this review will follow the framework for conducting a scoping study; Stage 1: identifying the research question, Stage 2: identifying relevant studies, Stage 3: study selection, Stage 4: charting the data and Stage 5: collating, summarizing and reporting the results.(27) Detail on each stage is provided.

Stage 1: Identification the research question

The key research questions outlined in *Table 1* provide focus to the aim of the review to improve understanding of how digital research methods are applied to hard-to-reach populations including identification of methods, potential challenges, and ethical concerns. To inform the search for marginalized, excluded and hidden populations which may not be defined by a term, a list of populations of interest have been included in data extraction framework in *Annex A.*

Table 1: Research questions

For peer review only - http://bmjopen.bmj.com/site/about/guidelines.xhtml

Research Questions
(1) How are digital health research methods applied to marginalized, excluded and hidden populations?
The secondary research questions focus on the strengths and limitations of the identified methods which include;<br>(1) What are digital research methods commonly applied to include or target hard-to-reach populations?<br>(2) What are challenges in recruitment, retention, and response rate in digitally designed studies for hard-to-reach populations?<br>(3) What were identified areas of ethical, safety and anonymity concerns in using digital research platforms for hard-to-reach populations?

Stage 2: Identifying relevant studies

The search strategy aims to be comprehensive in its inclusion of studies across different socio-economic and environmental determinants of health among hard-to-reach populations. The example evidence map is available in *Annex B.* The selection of health, social science, mental health, and climate/environmental research databases will be employed in this review.(28) The database, description and article limit can be found in *Table 2*.

While we primarily focus on peer-reviewed studies for this scoping review, we will also search grey literature, technical reports, and policy briefs produced by key agencies following a novel approach as set out by Enticott et al (e.g. the World Health Organisation, UNICEF, the Campbell Collaboration.(29) These reports, guidance, and sector best practice will complement our primary evidence mapping based on individual studies, to ensure the most complete view of evidence is achieved. The search strategy is available in the supplementary file.

Table 2: Databases and Sources

Database/Source	Focus	Article limit per search term combination
Published literature		
PubMed	Health and medical research	None
Cochrane Library	Relevant systematic reviews on digital health	None
PsycINFO	Mental health and social research	None
Google Scholar	All search terms	200
Greenfile	Impact of climate change on health	None
Grey literature		
Key technical agencies (World Health Organization, UNICEF etc.)	Hard-to-reach/marginalization population specific research guidance – digital and non-digital	50
Campbell Collaboration	Social Sciences	50

A Population, Concept, Context framework (PCC) for this review is illustrated in *Table 3.*(30) Various search terms will be included, namely common hard-to-reach populations, socio-economic and environmental determinants of health and names of various digital platforms and methods. These are in line with the draft data extraction framework. Completed search terms will be documented and shared in the final publication.

For peer review only - http://bmjopen.bmj.com/site/about/guidelines.xhtml

Table 3: Population, Concept, Context framework (PCC)

PCC	Definition	Example
Population	Hard-to-Reach	Homeless
Concept	Digital research methods	Online survey
Context	Health and social research	Mental Health

Stage 3: Study Selection

The review has established a wide inclusion criteria. The geographic scope of this research is global but studies are limited to those published in English. Inclusion and exclusion criteria can be found in *Table 4.* Rayyan and Endnote will be used to store all search results. The first reviewer will screen all titles and abstracts against the criteria. The second reviewer will screen 10% of these to validate the screening process for inclusion and exclusion criteria. The first reviewer will then import the full text of all included documents for detailed analysis.

Table 4: Inclusion and Exclusion Criteria

Theme	Inclusion Criteria	Exclusion Criteria
Geographic	All	None
Time	01/02/2012 to 01/02/2022	Before 2012
Language	English	All other languages
Populations	Keywords listed in *Table A* and search strategy in *Annex C*	Digital health studies which are not focused on hard-to-reach/marginalized populations
Type of literature	Published, peer-reviewed literature<br>Grey literature including technical reports and policy briefs that are endorsed by key agencies.	Literature which is not peer-reviewed<br>Unpublished literature which is not considered sector guidance including presentations, webinars, and case studies
Digital Application	Digital research approaches including digital data collection, use of apps to collect data, digital surveys, use of social media and other strategies, including using machine-learning analytical techniques.	Digital interventions such as remote consultations, digital health communications, social media health campaigns.<br><br>Digital information systems which are not part of a research design method will be excluded.

Stage 4: Charting the data

The data charting will occur using a google form collecting the recommended general information about the study according to the guidance for scoping reviews and expanding data collection through a data extraction framework into key study characteristics, including type of digital study, methodology and limitations. The data extraction framework, available in *Annex A* will be tested by both reviewers for accuracy and applicability and applied consistently across all included studies. The first reviewer (RB) will extract all data and the second reviewer (YSC) will review 10% of the articles to check for alignment. If misalignment is identified, the second reviewer will provide a full review of literature. The grey literature search will be performed by the first reviewer.

Stage 5: Collating, summarizing and reporting the results

For peer review only - http://bmjopen.bmj.com/site/about/guidelines.xhtml

The data collected in the charting exercise will be analyzed by both reviewers. The digital methods deployed will be documented with particular focus to how the method enabled inclusivity of the hard-to-reach population and followed ethical guidance for research. The limitations of the studies will be analyzed with a focus on research strength, ethics, replicability, community engagement, and feasibility. Quality of the reviewed studies will be assessed using checklists informed from Joanna Briggs Institute (JBI) (https://jbi.global/critical-appraisal-tools) for different types of studies. Additional analysis will include the identification of key research gaps and future research priorities at the intersection of digital health research and marginalized populations.

PATIENT AND PUBLIC INVOLVEMENT
No patient involved.

ETHICS AND DISSEMINATION
This scoping review does not require ethical approval as it reviews already available publications. The results of the scoping review will consist of peer-reviewed publications, presentations and knowledge mobilization activities including a lay summary posted via social media channels and production of policy brief.

AUTHOR AFFILIATIONS:
University of Oxford
Nuffield Department of Women's & Reproductive Health
1st Floor, Hayes House, 75 George Street
Oxford, OX1 2BQ, United Kingdom

University of Leicester
Centre for Environmental Health and Sustainability,
Department of Health Sciences,
University Road, LE1 7RH, Leicester, United Kingdom
University of Leicester

AUTHORS' CONTRIBUTIONS:
RB: conceived the initial idea, designed the review, drafted the manuscript. KR and SC: provided inputs to the study protocol and revised manuscript. All authors approved the manuscript for submission.

FUNDING STATEMENT:
This research was funded by the PEAK Urban programme, UKRI's Global Challenge Research Fund, Grant Ref: ES/P011055/1; supported by the National Institute for Health Research (NIHR) Oxford Biomedical Research Centre (BRC), the Oxford Martin school, University of Oxford and the British Heart Foundation grant ref: PG/18/65/33872. The views expressed are those of the authors and not necessarily those of the OMS, the UK National Health Service (NHS), the NIHR or the Department of Health and Social Care.

The funding organizations had no role in design or conduct of the study; collection, management, analysis, and interpretation of the data; preparation, review, or approval of the manuscript; or decision to submit the manuscript for publication.

YSC acknowledges support from National Institute for Health Research (NIHR) Health Protection Research Unit in Environmental Exposures and Health, a partnership between the

For peer review only

UK Health Security Agency, the Health and Safety Executive and the University of Leicester. The views expressed are those of the author(s) and not necessarily those of the NHS, the NIHR, the Department of Health and Social Care or the UK Health Security Agency.

COMPETING INTERESTS STATEMENT

The authors note there are no competing interests, financial or personal which have influenced this research.

REFERENCES

1. Lilford RJ, Oyebode O, Satterthwaite D, Melendez-Torres GJ, Chen YF, Mberu B, et al. Improving the health and welfare of people who live in slums. The Lancet. 2017 Feb 4;389(10068):559–70.
2. Snee H, Hine C, Morey Y, Roberts S, Watson H. Digital Methods as Mainstream Methodology: An Introduction. Digital Methods for Social Science [Internet]. 2016 [cited 2022 Jan 7];1–11. Available from: https://link.springer.com/chapter/10.1057/9781137453662_1
3. Gunasekeran DV, Tseng RMWW, Tham YC, Wong TY. Applications of digital health for public health responses to COVID-19: a systematic scoping review of artificial intelligence, telehealth and related technologies. npj Digital Medicine 2021 4:1 [Internet]. 2021 Feb 26 [cited 2021 Dec 12];4(1):1–6. Available from: https://www.nature.com/articles/s41746-021-00412-9
4. Rodriguez JA, Clark CR, Bates DW. Digital Health Equity as a Necessity in the 21st Century Cures Act Era. JAMA : the journal of the American Medical Association. 323(23):2381–2.
5. Keesara S, Jonas A, Schulman K. Covid-19 and Health Care's Digital Revolution. New England Journal of Medicine [Internet]. 2020 Jun 4 [cited 2022 Jan 7];382(23):e82. Available from: https://www.nejm.org/doi/full/10.1056/NEJMp2005835
6. Whitelaw S, Mamas MA, Topol E, C Van Spall HG. Applications of digital technology in COVID-19 pandemic planning and response. 2020; Available from: www.thelancet.com/
7. Hlatshwako TG, Shah SJ, Kosana P, Adebayo E, Hendriks J, Larsson EC, et al. Online health survey research during COVID-19. Lancet Digit Health [Internet]. 2021 Feb 1 [cited 2022 Jan 8];3(2):e76–7. Available from: http://www.ncbi.nlm.nih.gov/pubmed/33509387
8. Jung RG, di Santo P, Clifford C, Prosperi-Porta G, Skanes S, Hung A, et al. Methodological quality of COVID-19 clinical research. Available from: https://doi.org/10.1038/s41467-021-21220-5
9. Labrique A, Agarwal S, Tamrat T, Mehl G. OPEN WHO Digital Health Guidelines: a milestone for global health. Available from: https://doi.org/10.1038/s41746-020-00330-2
10. Crawford A, Serhal E. Digital Health Equity and COVID-19: The Innovation Curve Cannot Reinforce the Social Gradient of Health. Journal of Medical Internet Research [Internet]. 2020 Jun 2 [cited 2022 Jul 19];22(6). Available from: /pmc/articles/PMC7268667/
11. THE 17 GOALS | Sustainable Development [Internet]. [cited 2022 Jan 7]. Available from: https://sdgs.un.org/goals
12. Mude W, Oguoma VM, Nyanhanda T, Mwanri L, Njue C. Racial disparities in COVID-19 pandemic cases, hospitalisations, and deaths: A systematic review and meta-analysis.

For peer review only - http://bmjopen.bmj.com/site/about/guidelines.xhtml

Journal of Global Health [Internet]. 2021 [cited 2022 Jan 7];11:1–15. Available from: /pmc/articles/PMC8248751/
13. Jacques A, Ambrose H. Inequities During COVID-19. 2020 [cited 2022 Jan 7]; Available from: https://doi.org/10.1542/peds.2020-1501
14. Nebeker C, Torous J, Bartlett Ellis RJ. Building the case for actionable ethics in digital health research supported by artificial intelligence. BMC Medicine [Internet]. 2019 Jul 17 [cited 2021 Dec 11];17(1):1–7. Available from: https://bmcmedicine.biomedcentral.com/articles/10.1186/s12916-019-1377-7
15. Whitelaw S, Mamas MA, Topol E, C Van Spall HG. Applications of digital technology in COVID-19 pandemic planning and response. 2020 [cited 2022 Jan 7]; Available from: www.thelancet.com/
16. O'connor S, Hanlon P, O'donnell CA, Garcia S, Glanville J, Mair FS. Understanding factors affecting patient and public engagement and recruitment to digital health interventions: a systematic review of qualitative studies. 2016 [cited 2021 Dec 12]; Available from: http://www.crd.york.ac.uk/
17. Helena M, Velthoven V, Smith J, Wells G, Brindley D. Digital health app development standards: a systematic review protocol. BMJ Open [Internet]. 2018 [cited 2021 Dec 12];8:22969. Available from: https://www.
18. Giustini D, Ali SM, Fraser M, Kamel Boulos MN. Effective uses of social media in public health and medicine: a systematic review of systematic reviews. [cited 2021 Dec 12]; Available from: http://ojphi.org*10
19. Adjekum A, Blasimme A, Vayena E. Elements of Trust in Digital Health Systems: Scoping Review. J Med Internet Res 2018;20(12):e11254 https://www.jmir.org/2018/12/e11254 [Internet]. 2018 Dec 13 [cited 2021 Dec 12];20(12):e11254. Available from: https://www.jmir.org/2018/12/e11254
20. Soobiah C, Cooper M, Kishimoto V, Bhatia S, Scott T, Maloney S, et al. Identifying optimal frameworks to implement or evaluate digital health interventions: a scoping review protocol. BMJ Open [Internet]. 2020 [cited 2021 Dec 12];10:37643. Available from: http://bmjopen.bmj.com/
21. Edo-Osagie O, de La Iglesia B, Lake I, Edeghere O. A scoping review of the use of Twitter for public health research. Computers in Biology and Medicine. 2020 Jul 1;122:103770.
22. Bonevski B, Randell M, Paul C, Chapman K, Twyman L, Bryant J, et al. Reaching the hard-to-reach: a systematic review of strategies for improving health and medical research with socially disadvantaged groups [Internet]. 2014. Available from: http://www.biomedcentral.com/1471-2288/14/42
23. Shaghaghi A, Bhopal RS, Sheikh A. Approaches to Recruiting "Hard-To-Reach" Populations into Re-search: A Review of the Literature. Health Promotion Perspectives. 2011;1(2):86–94.
24. Thunberg S, Arnell L. Pioneering the use of technologies in qualitative research – A research review of the use of digital interviews. Int J Soc Res Methodol. :1–12.
25. Whitaker C, Stevelink S, Fear N. The use of Facebook in recruiting participants for health research purposes: A systematic review. J Med Internet Res. 19(8):e290–e290.

For peer review only - http://bmjopen.bmj.com/site/about/guidelines.xhtml

26. Nebeker C, Dunseath SE, Linares-Orozco R. A retrospective analysis of NIH-funded digital health research using social media platforms. Digital Health. 2020;6:TYPE=Issue|ARK=ark:/81055/vdc_100112557518.0x00...
27. Arksey H, O'Malley L. Scoping studies: towards a methodological framework. Int J Soc Res Methodol. 8(1):19–32.
28. Bramer W, Rethlefsen ML, Kleijnen J, Franco O. Optimal database combinations for literature searches in systematic reviews: A prospective exploratory study. Syst Rev. 6(1):245–245.
29. Enticott J, Buck K, Shawyer F. Finding "hard to find" literature on hard to find groups: A novel technique to search grey literature on refugees and asylum seekers. International Journal of Methods in Psychiatric Research. 2018 Mar 1;27(1).
30. Peters M, Godfrey C, McInerney P, Munn Z, Trico A, Khalil H. Chapter 11: Scoping Reviews. JBI Manual for Evidence Synthesis. 2020;

For peer review only

For peer review only - http://bmjopen.bmj.com/site/about/guidelines.xhtml

Supplementary Files for "Researching the hard-to-reach: a scoping review protocol of digital health research in hidden, marginal, and excluded populations"

A. Documented search strategy

Contents

I. Summary of search methods and approach
II. Pubmed
III. Cochrane Review
IV. Pysch Info
V. Greenfile
VI. Google Scholar
VII. Grey literature

I. Summary of search methods and approach

– Screening for inclusion of published literature performed by first reviewer from February 1st to March 1st 2022
– Screening for inclusion planned by second reviewer in August 2022
– Data extraction from the first review occurred in parallel following the testing of the data extraction framework by both reviewers.

Summary of search strategy method:

Database/Source	Focus	Article limit per search term combination
Published literature		
PubMed	Health and medical research	None
Cochrane Library	Relevant systematic reviews on digital health	None
PsycINFO	Mental health and social research	None
Google Scholar	All search terms	200
Greenfile	Impact of climate change on health	None
Grey literature		
Websites of key technical agencies (World Health Organization, UNICEF)	Hard-to-reach/marginalization population specific research guidance – digital and non-digital	50
Campbell Collaboration	Social Science	None

II. PubMed [Search performed February 1 2022]

Concept 1: Digital

Keywords: "Digital health research"[TIAB] OR "Digital Survey*"[TIAB] OR "Digital Epidemiology" [TIAB] OR "Online survey*"[TIAB] *OR* "Mobile technology"[TIAB] OR "Health application*"[TIAB] OR "Health app"[TIAB] OR "Interactive Voice Response" [TIAB] OR "SMS" [TIAB] OR "Whatsapp"

For peer review only - http://bmjopen.bmj.com/site/about/guidelines.xhtml

[TIAB] OR “Facebook”[TIAB] OR “Twitter”[TIAB] OR “smartphone*”[TIAB] OR “Auto-photography”[TIAB] OR “Video”[TIAB] OR “Photography”[TIAB] OR “Smartwatch*”[TIAB]

MH: “Digital Technology*”[MH] OR “Cell phone”[MH] OR “Mobile Applications”[MH] OR “Social Media”[MH] OR “Digital Technology”[MH] OR “Wearable electronic devices”[MH]

AND

Concept 2: Hard-to-Reach Populations

Keywords: “Hard-to-reach”[TIAB] OR “Marginalized”[TIAB] OR “Refugee*”[TIAB] OR “Migrant*”[TIAB] OR “Vulnerable”[TIAB] OR “Internally Displaced Person*”[TIAB] OR “Slum Resident*”[TIAB] OR “Slumdweller*”[TIAB] OR “Homeless”[TIAB] OR “Housing insecure”[TIAB] OR “Illiterate”[TIAB] OR “Disability”[TIAB] OR “Remote-Rural”[TIAB] OR “Nomadic”[TIAB] OR “Indigenous”[TIAB] OR “Immigrant*”[TIAB] OR “Prisoner*”[TIAB] OR “Low-income”[TIAB] OR “LGTBQ”[TIAB] OR “Injecting drug-users”[TIAB]

MH: “Health Disparity, Minority and Vulnerable Populations”[MH] OR "Homeless Persons"[MH] OR "Opioid-Related Disorders"[MH] OR "Refugees"[MH] OR "Prisoners"[MH] OR "Poverty Areas"[MH] OR "Transients and Migrants"[MH] OR "Health Services for Transgender Persons"[MH] OR "Vulnerable Populations"[MH] OR "Social Vulnerability"[MH] OR "Intellectual Disability"[MH] OR "Disabled Persons"[MH] OR "Social Discrimination"[MH] OR "Persons with Mental Disabilities"[MH]

AND

Concept 3: Health and social-determinants of health

Keywords: “Health”[TIAB] OR “Nutritional Status”[TIAB] OR “Nutrition”[TIAB] OR “Mental Health”[TIAB] OR “Unemployment”[TIAB] OR “Food insecure”[TIAB] OR “Discrimination”[TIAB] OR “Conflict”[TIAB] OR “Natural Disaster”[TIAB] OR “Flooding”[TIAB] OR “Pollution”[TIAB]

MH: "Health"[MH] OR "Social Determinants of Health"[MH] OR "Environmental Pollution"[MH] OR “Natural Disasters”[MH]

Documented Search Strategy

**Final Search –** 4,966, limit to 10 years – 4,499. Search performed Feb 1, 2022	(("Digital health research"[TIAB] OR "Digital Survey*"[TIAB] OR "Digital Epidemiology" [TIAB] OR "Online survey*"[TIAB] OR "Mobile technology"[TIAB] OR "Health application*"[TIAB] OR "Health app"[TIAB] OR "Interactive Voice Response" [TIAB] OR "SMS" [TIAB] OR "Whatsapp" [TIAB] OR "Facebook"[TIAB] OR "Twitter"[TIAB] OR "smartphone*"[TIAB] OR "Auto-photography"[TIAB] OR "Video"[TIAB] OR "Photography"[TIAB] OR "Smartwatch*"[TIAB] OR "Digital Technology*"[MH] OR "Cell phone"[MH] OR "Mobile Applications"[MH] OR "Social Media"[MH] OR "Digital Technology"[MH] OR "Wearable electronic devices"[MH]) AND ("Hard-to-reach"[TIAB] OR "Marginalized"[TIAB] OR "Refugee*"[TIAB] OR "Migrant*"[TIAB] OR "Vulnerable"[TIAB] OR "Internally Displaced Person*"[TIAB] OR "Slum Resident*"[TIAB] OR

For peer review only

	"Slumdweller*"[TIAB] OR "Homeless"[TIAB] OR "Housing insecure"[TIAB] OR "Illiterate"[TIAB] OR "Disability"[TIAB] OR "Remote-Rural"[TIAB] OR "Nomadic"[TIAB] OR "Indigenous"[TIAB] OR "Immigrant*"[TIAB] OR "Prisoner*"[TIAB] OR "Low-income"[TIAB] OR "LGTBQ"[TIAB] OR "Injecting drug-users"[TIAB] OR "Health Disparity, Minority and Vulnerable Populations"[MH] OR "Homeless Persons"[MH] OR "Opioid-Related Disorders"[MH] OR "Refugees"[MH] OR "Prisoners"[MH] OR "Poverty Areas"[MH] OR "Transients and Migrants"[MH] OR "Health Services for Transgender Persons"[MH] OR "Vulnerable Populations"[MH] OR "Social Vulnerability"[MH] OR "Intellectual Disability"[MH] OR "Disabled Persons"[MH] OR "Social Discrimination"[MH] OR "Persons with Mental Disabilities"[MH])) AND ("Health"[TIAB] OR "Nutritional Status"[TIAB] OR "Nutrition"[TIAB] OR "Mental Health"[TIAB] OR "Unemployment"[TIAB] OR "Food insecure"[TIAB] OR "Discrimination"[TIAB] OR "Conflict"[TIAB] OR "Natural Disaster"[TIAB] OR "Flooding"[TIAB] OR "Pollution"[TIAB] OR "Health"[MH] OR "Social Determinants of Health"[MH] OR "Environmental Pollution"[MH] OR "Natural Disasters"[MH])
1 February 2022<br>• Pubmed search exported to endnote<br>• Endnote export to RIS file<br>• 4,499 references uploaded to Rayyan	

III. Cochrane Reviews – Search performed 1 March 2022

Due to the broad reach of the complimentary databases in this review, only Cochrane Reviews were included.

**Search Term for Title and Abstract.**	**Total Cochrane Reviews**
"Digital Health"	61
"Digital Technology"	30
"Hard-to-Reach"	3
A total of 91 Cochrane reviews were added to Rayyan.	

IV. PSYCH Info – search performed 20 February 2022

According to the methodology, there was no article limit, although only articles from 2012 were included.

**Search Terms combinations**	**Total articles**
(Digital) OR (Mobile technology)	86,159
(Vulnerable) OR (Hard-to-Reach)	89,864

For peer review only

(Health) OR (Climate)	1,963,823
**Final Search Strategy:** "Digital or Mobile technology AND vulnerable or hard-to-reach AND health or climate"	2,307

V. Google Scholar – Search performed on 28 February 2022

According to the methodology, 200 articles were the limit given the potential for duplication with other databases. Only articles from 2012 were included.

Search Terms combinations	Total articles
Vulnerable "digital health"	200

VI. Greenfile – Search performed on 20 February 2022

Search Terms combinations	Total articles
"vulnerable populations or groups or people" AND "digital technology or web technology or software technology or virtual technology"	17

Duplications

Following the compilation of all database results, 401 duplicates were identified by Endnote and Rayyan.

VII. Grey Literature

A grey literature search will focus on guidelines, reviews and commentaries related to the progress of standard setting and best practice for digital research inclusion. The review will include sector Guidance issued from the following institutions: the World Health Organisation, UNICEF and the Campbell Collaboration. References of key studies included in the review will also be considered. The articles identified in the grey literature search will be used to inform the data extraction framework and the analysis. This work is ongoing as of July 2022, informed by the feedback of the reviewers.

Planned search strategy for grey literature:

Search Terms	Database or Website	Document Limit
"Digital Health", "Digital Technology", "Hard-to-Reach"	Cochrane Collaboration	50
"Digital Health", "Digital Technology", "Hard-to-Reach"	The World Health Organisation, UNICEF	50
"Digital Health", "Digital Technology", "Hard-to-Reach" + "Guidance"	Google	50

For peer review only

B. Summary of inclusion and exclusion for published literature

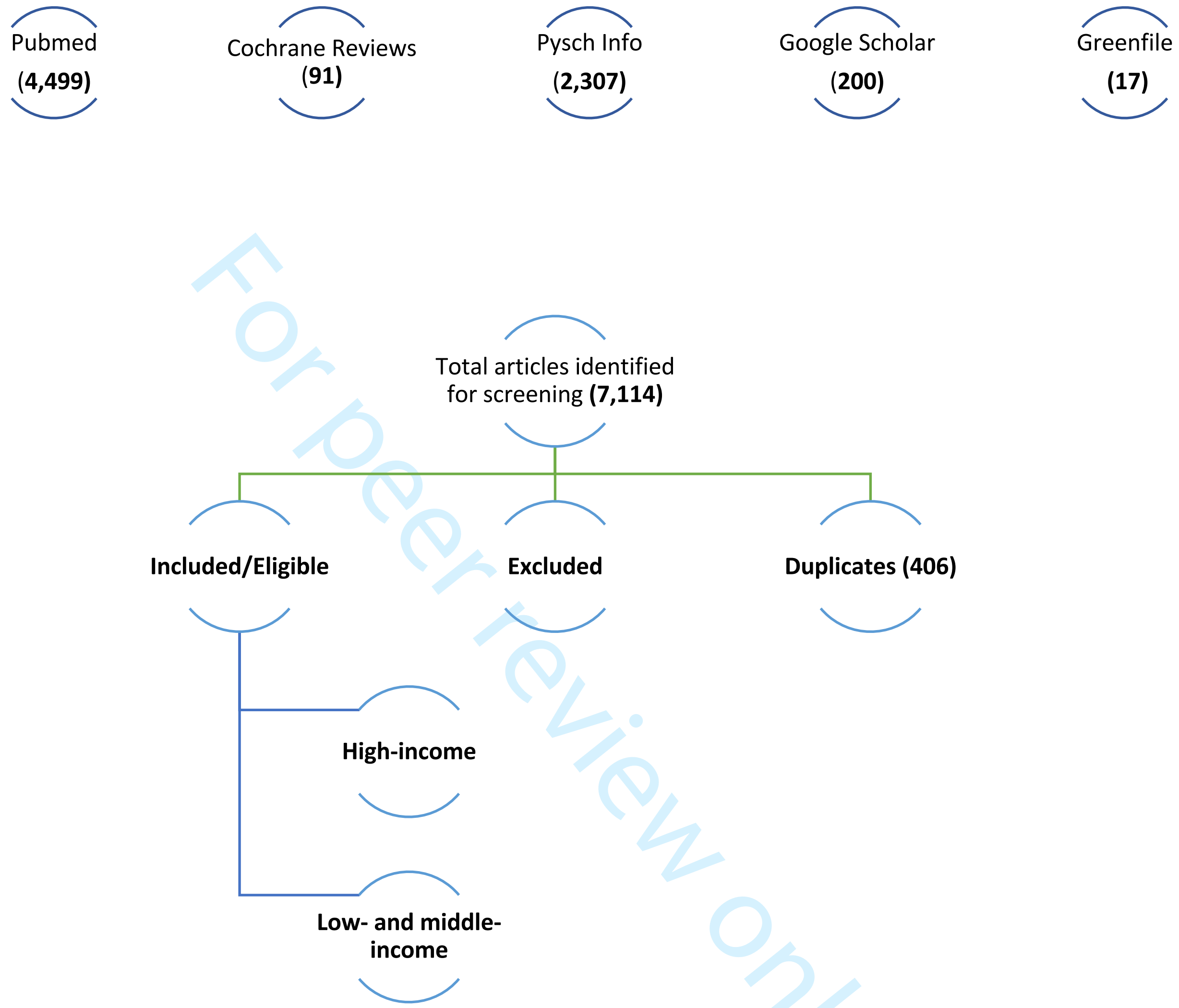

For peer review only

An evidence map was developed to plan the review following guidance of a systematic review by Miake-Lye.(26)

C. Data Extraction Framework

This framework has been adapted from the Chapter 11.3.7.3. of the data extraction guidance from JBI.(25) This is a draft will be adapted and iterated to a final version in line with the iterative process permitted in the Arksey and O' Malley methodological framework and will be documented in the final publication.(27)

Theme	Fields of data collection/categorization
Article source and authorship	Authors<br>Title<br>Journal/Source<br>Year of publication<br>Country<br>High-income country<br>Middle-income country<br>Low-income country
Populations of interest (more than one selection, permitted)	Refugee<br>Migrant<br>Internally Displaced Person<br>Informal Settlement Resident<br>Homeless<br>Low-literacy<br>Disability including physical, developmental, behavioral or emotional and sensory-impaired disorders<br>Remote-rural<br>Nomadic<br>LGBTQ+<br>Low-income<br>Minority<br>Youth *<br>Elderly*<br>Other _____<br>*These two populations are not included alone but with a secondary population. However, due to the presence of a large number of studies related to these populations they have been included for the analysis,
Type of digital research and digital platform (more than one selection, permitted)	Big Data (Machine learning, AI)<br>Digital Survey<br>Digital Epidemiology<br>Online Survey<br>Interactive Voice Response<br>SMS<br>WhatsApp<br>Facebook Survey Twitter<br>App (application, bespoke)<br>Smartphone<br>Auto-photography<br>Video

For peer review only

	Photography<br>Wearable<br>Other ___
Research study focus	Health<br>COVID-19 (y/n)<br>Nutritional status<br>Mental Health<br>Social-determinants of health;(28)<br>• Income and social protection<br>• Education<br>• Unemployment and job insecurity<br>• Working life conditions<br>• Food insecurity<br>• Housing, basic amenities and the environment<br>• Early childhood development<br>• Social inclusion and nondiscrimination<br>• Structural conflict<br>• Access to affordable health services of decent quality<br>Physical environmental determinants of health (air-quality, pollution)<br>Climate-related factors such as flooding and heat
Research category	Qualitative<br>Quantitative
Methods and limitations	Recruitment (multiple selections allowed)<br>• Social Media<br>• In-person<br>• snowball sampling<br>• respondent-driven sampling (RDS)<br>• indigenous field-worker sampling (IFWS)<br>• facility-based sampling (FBS)<br>• targeted sampling (TS)<br>• time-location (space) sampling (TLS)<br>• conventional cluster sampling (CCS)<br>• capture re-capture sampling (CR) (19)<br>• Other<br><br>Response rate<br>Use of incentives<br>Anonymity<br>Ethics<br>Presence of community engagement
Quality	Considerations for a rapid quality review of included studies will be developed based on checklists from Joanna Briggs Institute (JBI) (https://jbi.global/critical-appraisal-tools)

For peer review only

Preferred Reporting Items for Systematic reviews and Meta-Analyses extension for Scoping Reviews (PRISMA-ScR) Checklist

SECTION	ITEM	PRISMA-ScR CHECKLIST ITEM	REPORTED ON PAGE #
**TITLE**			
Title	1	Identify the report as a scoping review.	1
**ABSTRACT**			
Structured summary	2	Provide a structured summary that includes (as applicable): background, objectives, eligibility criteria, sources of evidence, charting methods, results, and conclusions that relate to the review questions and objectives.	1
**INTRODUCTION**			
Rationale	3	Describe the rationale for the review in the context of what is already known. Explain why the review questions/objectives lend themselves to a scoping review approach.	2, 3
Objectives	4	Provide an explicit statement of the questions and objectives being addressed with reference to their key elements (e.g., population or participants, concepts, and context) or other relevant key elements used to conceptualize the review questions and/or objectives.	3
**METHODS**			
Protocol and registration	5	Indicate whether a review protocol exists; state if and where it can be accessed (e.g., a Web address); and if available, provide registration information, including the registration number.	3
Eligibility criteria	6	Specify characteristics of the sources of evidence used as eligibility criteria (e.g., years considered, language, and publication status), and provide a rationale.	5
Information sources*	7	Describe all information sources in the search (e.g., databases with dates of coverage and contact with authors to identify additional sources), as well as the date the most recent search was executed.	5
Search	8	Present the full electronic search strategy for at least 1 database, including any limits used, such that it could be repeated.	Annex C
Selection of sources of evidence†	9	State the process for selecting sources of evidence (i.e., screening and eligibility) included in the scoping review.	5
Data charting process‡	10	Describe the methods of charting data from the included sources of evidence (e.g., calibrated forms or forms that have been tested by the team before their use, and whether data charting was done independently or in duplicate) and any processes for obtaining and confirming data from investigators.	6
Data items	11	List and define all variables for which data were sought and any assumptions and simplifications made.	NA at this stage
Critical appraisal of individual sources of evidence§	12	If done, provide a rationale for conducting a critical appraisal of included sources of evidence; describe the methods used and how this information was used in any data synthesis (if appropriate).	NA at this stage
Synthesis of results	13	Describe the methods of handling and summarizing the data that were charted.	6

For peer review only - http://bmjopen.bmj.com/site/about/guidelines.xhtml

SECTION	ITEM	PRISMA-ScR CHECKLIST ITEM	REPORTED ON PAGE #
**RESULTS**			
Selection of sources of evidence	14	Give numbers of sources of evidence screened, assessed for eligibility, and included in the review, with reasons for exclusions at each stage, ideally using a flow diagram.	NA at this stage, Draft in Annex B.
Characteristics of sources of evidence	15	For each source of evidence, present characteristics for which data were charted and provide the citations.	NA at this stage
Critical appraisal within sources of evidence	16	If done, present data on critical appraisal of included sources of evidence (see item 12).	"
Results of individual sources of evidence	17	For each included source of evidence, present the relevant data that were charted that relate to the review questions and objectives.	"
Synthesis of results	18	Summarize and/or present the charting results as they relate to the review questions and objectives.	"
**DISCUSSION**			
Summary of evidence	19	Summarize the main results (including an overview of concepts, themes, and types of evidence available), link to the review questions and objectives, and consider the relevance to key groups.	NA at this stage
Limitations	20	Discuss the limitations of the scoping review process.	"
Conclusions	21	Provide a general interpretation of the results with respect to the review questions and objectives, as well as potential implications and/or next steps.	"
**FUNDING**			
Funding	22	Describe sources of funding for the included sources of evidence, as well as sources of funding for the scoping review. Describe the role of the funders of the scoping review.	6

JBI = Joanna Briggs Institute; PRISMA-ScR = Preferred Reporting Items for Systematic reviews and Meta-Analyses extension for Scoping Reviews.

* Where *sources of evidence* (see second footnote) are compiled from, such as bibliographic databases, social media platforms, and Web sites.

† A more inclusive/heterogeneous term used to account for the different types of evidence or data sources (e.g., quantitative and/or qualitative research, expert opinion, and policy documents) that may be eligible in a scoping review as opposed to only studies. This is not to be confused with *information sources* (see first footnote).

‡ The frameworks by Arksey and O'Malley (6) and Levac and colleagues (7) and the JBI guidance (4, 5) refer to the process of data extraction in a scoping review as data charting.

§ The process of systematically examining research evidence to assess its validity, results, and relevance before using it to inform a decision. This term is used for items 12 and 19 instead of "risk of bias" (which is more applicable to systematic reviews of interventions) to include and acknowledge the various sources of evidence that may be used in a scoping review (e.g., quantitative and/or qualitative research, expert opinion, and policy document).

From: Tricco AC, Lillie E, Zarin W, O'Brien KK, Colquhoun H, Levac D, et al. PRISMA Extension for Scoping Reviews (PRISMAScR): Checklist and Explanation. Ann Intern Med. 2018;169:467–473. doi: 10.7326/M18-0850.

For peer review only - http://bmjopen.bmj.com/site/about/guidelines.xhtml
